# Establishment and observation of a new and ideal reversible model of PUUO

**DOI:** 10.1080/0886022X.2016.1256318

**Published:** 2016-11-15

**Authors:** Wei Zhu, Xi Zheng, Chunming Jiang, Haifeng Huang, Wei Wang, He Liu, Wei Jiang, Lin Yang, Shengjie Zhang, Miao Zhang, Dalong Zhu, Xiang Yan

**Affiliations:** a Department of Nephrology, Drum Tower Clinical Medical School, Nanjing Medical University, Nanjing, PR China;; b Department of Urology, Drum Tower Hospital, Medical School of Nanjing University, Nanjing, PR China;; c Institution of Internal Medicine, Drum Tower Clinical Medical School, Nanjing Medical University, Nanjing, PR China

**Keywords:** Partial unilateral ureteral obstruction, hydronephrosis, new animal model, tee joint, reversible

## Abstract

**Objective:** We established a novel procedure to generate a reversible partial unilateral ureteral obstruction (PUUO) in rabbit. The method allows us to reliably measure the degree of ureteral obstruction in live animals, and thus could be a useful tool for studying kidney diseases.

**Methods:** Thirty rabbits of clean grade were divided randomly into sham control group and obstruction (PUUO) group. Each rabbit in this study received the same blocking surgery, in which the upper ureter was curvilinearly incised and inserted with two F6 ureteral catheters that were connected with a tee joint valve. Ureteral obstruction was created and released through the valve adjustment. Serum creatinine and ultrasonographic measurements were performed preoperatively, on the fifth and tenth days after obstruction surgery, and on the 10th and 20th day, respectively, after the relief of the obstruction. Pathological measurements were taken in two randomly chosen rabbits of each group on the 10th day after surgery and on the 20th day after obstruction relief.

**Results:** Data showed that the serum creatinine went transiently up and down in the early days and then remained a little bit higher in the following days after obstruction surgery. The morphology in obstructed kidney changed significantly on the 10th day postoperatively, compared to the sham control group. The obvious differences were also observed in pathology tests. After the relief of obstruction, the volume of renal pelvis (V), renal cortical thickness (RCT), and pathological impairment were partially reversed.

**Conclusions:** Those findings indicate our procedure generate a successful and reversible PUUO animal model. It is a reliable and simple procedure for generating an animal model for reversible PUUO. The feasibility and significance of the new method was confirmed through ultrasonographic and pathological results.

## Introduction

The obstructive nephropathy is caused by the lesion of an occlusive urinary track and/or its neighboring structures. Ureteral obstruction ultimately results in hydronephrosis and loss of renal function. The unilateral ureteral obstruction (UUO) model sits at the interface between acute kidney injury (AKI) and chronic kidney disease (CKD). The acute nature of the insult leads to AKI. Persistence of the obstruction leads to histological features of CKD in one to two weeks.^1^^,^^2^ Clinically, the patients of partial unilateral ureteral obstruction (PUUO) have taken a great part of all the sufferers in unilateral ureteral obstruction. Experimental PUUO is an animal model of human disease, which reproduces not only ureteral obstruction induced by kidney injury in human, but also models several key pathogenic processes of AKI and CKD, including tubular cell injury, interstitial inflammation and fibrosis.^1^ For these reasons, the PUUO model is a popular experimental model for obstructive nephropathy. However, the reversible PUUO models in this manuscript can mimic the entire lesion or recovery process of obstructive nephropathy in clinical settings.

At present, there are a few surgical methods to create PUUO in animals, such as the Ulm and Miller’s psoas muscle approach,^3^ Cheng and Chevalier’s tube approach and Harada’s partial ligation approach.^4^^,^^5^^,^^6^ However, the models mentioned above had their disadvantages. Most models could not achieve the goal of accurate quantification of occlusion, and some required a second operation on the same spot for occlusion relief, which is technically challenging and hard for tissue recovery.^3^^,^^4^^,^^5^^,^^6^

This study was designed to develop a new and reliable method to establish an ideal animal model of partial unilateral ureteral obstruction (PUUO) in rabbits. We surgically inserted an adjustable tee joint within ureteral tract to control the obstruction level accurately. Our PUUO model is the first animal model that has been successfully demonstrated to be reversible, quantifiable and obviate a second operation.

## Materials and methods

### Animals and groups

The experiment was carried out in the Animal Center of Affiliated Drum Tower Hospital, Nanjing University Medical School. All the rabbits were of clean grade, aged between 10 and 16 weeks, and weighing between 2500 and 3000 g. Thirty adult male rabbits of clean grade were divided randomly into two groups (15 each): 0% UUO group (sham group) and 50% PUUO group.

### Establishment of the animal model (retroperitoneal approach to the ureter)

**Figure 1.**
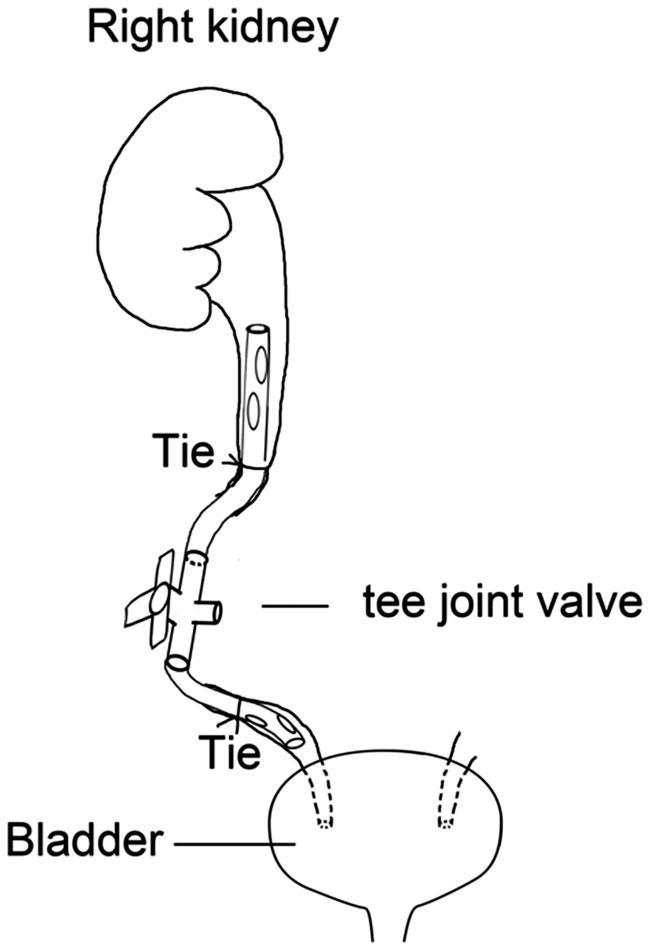
The operative model of PUUO. Ureteral stumps were implanted and fastened by two F6 ureteral catheters connected by a tee joint valve.

**Figure 2.**
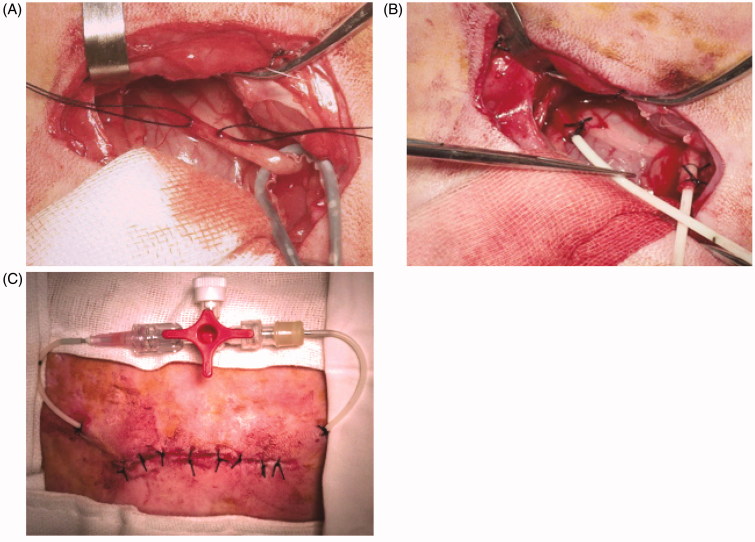
The operative procedure for reversible PUUO. (A) The right ureter was exposed. (B) Two F6 ureteral catheters were implanted and fastened into ureteral stumps. (C) Two F6 ureteral catheters were connected by a tee joint valve.

The rabbit was anesthetized with intramuscular ketamine (20 mg/kg) and placed in a prone position. It was draped in a usual sterile sheet after the skin preparation and sterilization. After having the parallel incision to the spine below the right kidney, anatomic layers were separated by blunt dissection and the upper ureter was exposed. Curvilinear incision was made on the upper ureter. Both ureteral stumps were implanted and fastened by two F6 ureteral catheters connected by a tee joint valve (Figure 1). Musculoaponeurotic layers were closed with a 4/0 absorbable suture and the skin was closed with a 5/0 suture (Figure 2). One dose of Penicillin (800,000 IU) was administered daily postoperatively. Operative incision was sterilized daily. The animals were allowed free access to water and food postoperatively.

All rabbits in this study underwent the same surgical procedure. In the sham control group, the implanted valve was open (no obstruction) entirely, while the ureteral valve was half-close to generate 50% obstruction in the PUUO group. Since the anatomic and pathological processes take place in 1–2 weeks postoperatively, the 10th day was defined as the checkpoint for model success.^7^^,^^8^ We relieved the obstruction in PUUO group at the 10th day if hydronephrosis existed. Fifteen rabbits from the PUUO group were randomly divided into relieved PUUO (RUUO) group, and continuous PUUO (CPUUO) group on the 10th day after surgery.

### Observation methods

#### Serum creatinine measurements

Blood samples were drawn from the ear vein. Samples from all rabbits were obtained to measure serum creatinine preoperatively and on the 5th and 10th days after first surgery, as well as the 10th and 20th day after relief of the obstruction. The baseline and serum creatinine at the 5th and 10th days were compared. The comparison of serum creatinine in RUUO group from different days and the differences between RUUO and CPUUO group were taken into plan.

#### Ultrasonographic measurements

The ultrasonographic examinations were performed by Color Doppler Imagining BK 2202UV (Denmark) with sector probe of 12 MHz. Renal cortical thickness (RCT), Longitudinal diameter (L), anteroposterior pelvis diameter (APD) and transverse diameter (T) were measured for each time. The volume of renal pelvis were calculated as Bartrum described (V = A × L × T × 0.523). The records were kept for reference values before surgery. On the 5th and 10th days after surgery, as well as on the 10th and 20th day after relief of the obstruction, the animals were carried to ultrasonography center for the ultrasonographic examinations and sedated with low-dose intramuscular ketamine administration before the examination.

#### Pathological measurements (hematoxylin-eosin stain)

Two rabbits of each group were chosen randomly for pathological examination on the 10th day after surgery and on the 20th day after obstruction relief, respectively. At sacrifice, the right kidney in rabbit were fixed with 4% paraformaldehyde, and embedded into paraffin. Paraffin-embedded sections were cut into 5 μm thickness, and stained with hematoxylin and eosin.

### Statistical analysis

The statistical analysis on the experimental results was conducted with the SPSS software (Chicago, IL). Quantitative data were expressed as mean ± standard deviation. For all analyses, a *p* values lower than .05 was accepted as statistically significant.

## Results

One rabbit in the sham control group and two rabbits in the PUUO group developed pyonephrosis. Intestinal injury unfortunately happened in one rabbit of each group, respectively, hence excluded from the study.

### Serum creatinine measurements

There was no significant difference of the pre-operative serum creatinine in the PUUO (111.15 ± 12.36 μM) and sham control group (114.16 ± 10.38 μM) (*p* > .05). Creatinine levels of the PUUO group (128.44 ± 12.28 μM) went upward and reached significance compared to the sham control group (115.41 ± 11.00 μM) on the 5th day postoperatively. However, the creatinine concentration in the PUUO group showed a decreasing trend in the following days. There was no significant difference in the 10th day between the PUUO (118.14 ± 13.28 μM) and shame control group (114.57 ± 9.372 μM), suggesting that the contralateral kidney might compensate for the obstruction (Table 1). After the relief of obstruction, the creatinine concentration in RUUO group on the 10th day and 20th day neither showed difference from that in the CPUUO group (*p* > .05) (Table 4), nor from the concentration before relief of obstruction (*p* > .05) (Table 3).

**Table 1.** The Mean ± standard deviation values of the creatinine.

	Creatinine concentration (umol/L)		
	Sham control group (*n* = 13)	PUUO group (*n* = 12)	*p* Values
Preoperative	114.16 ± 10.38	111.15 ± 12.36	>.05
Postoperative 5th day	115.41 ± 11.00	128.44 ± 12.28	<.05
Postoperative 10th day	114.57 ± 9.37	118.14 ± 13.28	>.05

### Ultrasonographic measurements

**Figure 3.**
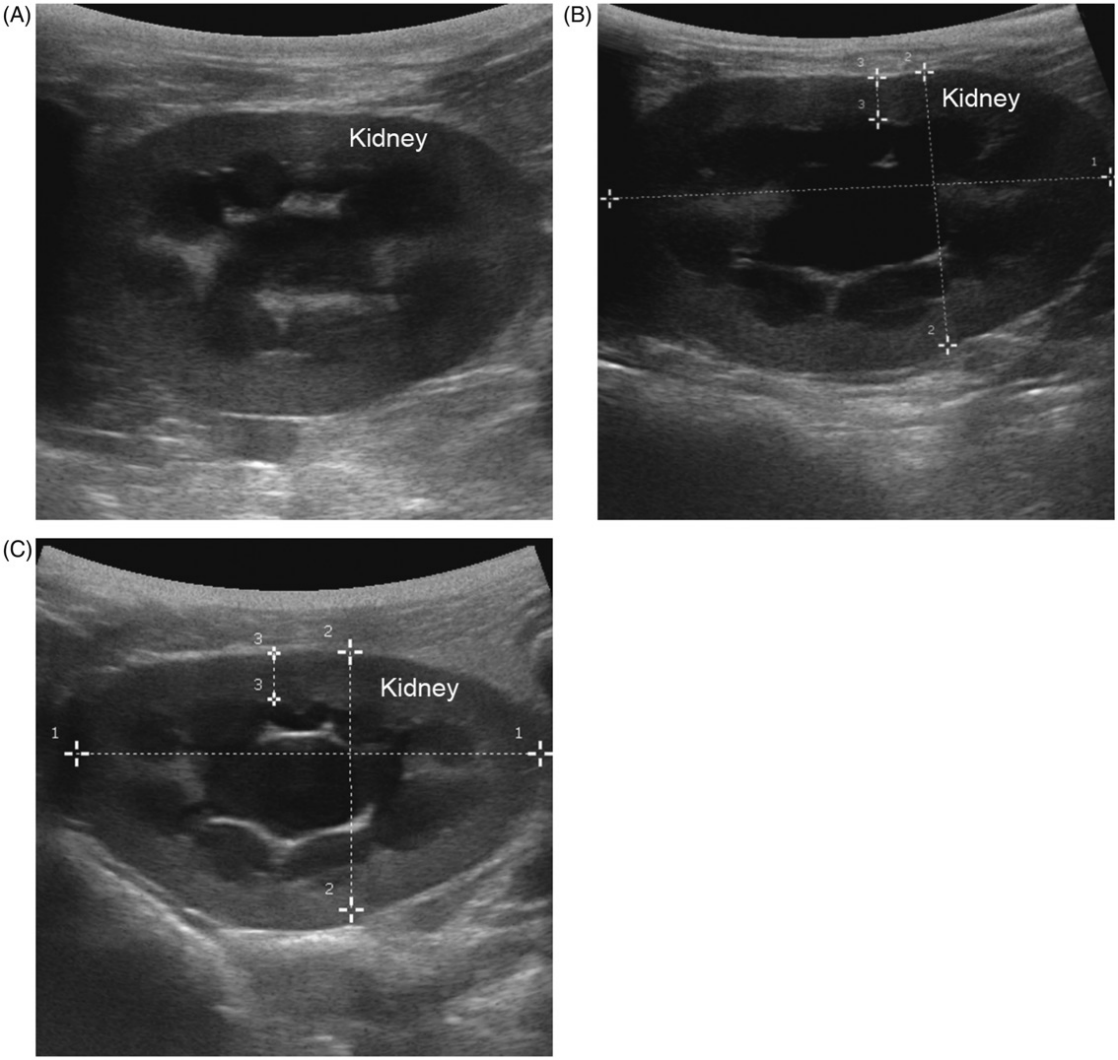
Postoperative ultrasonography of the kidney. (A) Ultrasonography of kidney in shame control group on the 10th day postoperatively. (B) Ultrasonography of kidney in PUUO group on the 10th day. The RCT decreased, while V increased into hydronephrosis. (C) Ultrasonography of kidney in RUUO group on the 20th day after relief.

Our data showed that the renal pelvis volume (V) and renal cortical thickness (RCT) have no significant difference in PUUO group and sham control group pre-operative (*p* > .05) (Table 2). Similar to the value of RCT, the value of V had no statistical significance in the sham control group preoperatively and post-operatively (1.09 ± 0.13, 1.18 ± 0.15, 1.21 ± 0.17cm3, respectively) (*p* > .05), suggesting that the sham operation had no harmful influences on the renal. The V was increased gradually after the obstructive operation, while RCT decreasing. The V reached significant difference on the 5th and 10th day in the PUUO group (3.51 ± 0.53, 8.57 ± 0.67cm^3^, respectively) compared to the sham control group (1.18 ± 0.15, 1.21 ± 0.17cm^3^, respectively) (*p* < .05), while the RCT reached significant difference on the 10th day (Table 2, Figure 3), indicating that the PUUO model was successfully established. After the obstruction was relieved, the V in the RUUO group decreased, while RCT increased, compared to the CPUUO group and the data before relief (*p* < .05, respectively) (Tables 3 and 4). These results showed the impact on kidney architecture after ureteral obstruction could be reversible.

**Table 2.** The mean ± standard deviation values of the V and RCT.

	Sham control group (*n* = 13)	PUUO group (*n* = 12)	*p* Values
Preoperative			
V (cm^3^)	1.09 ± 0.13	1.21 ± 0.21	>.05
RCT (mm)	4.45 ± 0.31	4.49 ± 0.28	>.05
Postoperative 5th day			
V (cm^3^)	1.18 ± 0.15	3.51 ± 0.53	<.01
RCT (mm)	4.44 ± 0.32	4.21 ± 0.27	>.05
Postoperative 10th day			
V (cm^3^)	1.21 ± 0.17	8.57 ± 0.67	<.01
RCT (mm)	4.42 ± 0.31	3.91 ± 0.33	<.01

**Table 3.** The mean ± standard deviation values of the creatinine, V and RCT in CPUUO and RUUO group.

	CPUUO group (*n* = 5)	RUUO group (*n* = 5)	*p* Values
10th day after relief			
Creatinine concentration (μM)	120.35 ± 10.49	125.09 ± 12.29	>.05
V (cm^3^)	9.95 ± 0.79	6.34 ± 0.52	<.05
RCT (mm)	3.55 ± 0.32	4.12 ± 0.33	<.05
20th day after relief			
Creatinine concentration (μM)	122.57 ± 8.56	123.26 ± 12.62	>.05
V (cm^3^)	13.37 ± 1.05	4.33 ± 0.38	<.05
RCT (mm)	3.27 ± 0.34	4.36 ± 0.35	<.05

**Table 4.** The mean ± standard deviation values of the creatinine, V and RCT in RUUO group (*n* = 5).

	Before relief	10th day after relief	20th day after relief
Creatinine(umol/L)	124.71 ± 11.97	125.09 ± 12.29	123.26 ± 12.62
V (cm^3^)	8.77 ± 0.76	6.34 ± 0.52^a^	4.33 ± 0.38^a^
RCT (mm)	4.01 ± 0.35	4.12 ± 0.33^a^	4.36 ± 0.35^a^

^a^(*p* < .05) Compared to data before relief of the obstruction.

### Pathological examination

**Figure 4.**
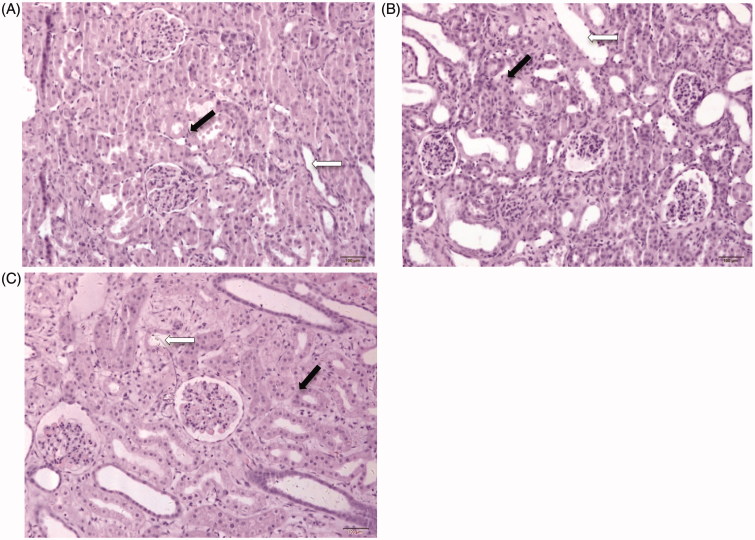
Postoperative kidney sections with hematoxylin and eosin (HE) staining. The hollow arrows represent the renal tubules. The solid arrows represent the renal interstitial. (A) HE for kidney in sham control group on the 10th day (200× folds). (B) HE staining for kidney in PUUO group on the 10th day (200× folds). Renal interstitial broadening and dilated tubules. (C) HE for kidney in RUUO group on the 20th day after relief (200× folds). Renal interstitial and tubules partially recovered.

Obvious renal interstitial broadening and tubules dilated were observed in renal sections after HE staining in the PUUO group on the 10th day, compared to the sham control group (Figure 4). This pathological change indicates the hydronephrosis. The pathological changes in the obstructed kidney were partially reversed on the 20th day in the RUUO group. These results indicate that kidney impairment can be induced after obstruction and repaired after the relief of obstruction.

## Discussion

PUUO represented a common clinical entity.^2^ In PUUO, when urine ejection from the renal pelvis to the ureter is curtained, kidney backpressure increases followed by hydronephrosis and progressive deterioration of kidney function. Timely relief of obstruction has always been the first priority for the treatment of obstructive nephropathy. The kidney lesion could be partially or completely rescued with timely relief and reducing the intrapelvic pressure. However, the research on intervening measurements and prediction of kidney prothetic capability after relief are rare to date. There are limited options for most surgeons or physicians to improve renal activity after relief of the obstruction clinically. Therefore, a reliable and easily reversible PUUO model is the key foundation for further studies on obstructive mechanism, prognosis and treatment.

Our study successfully established a PUUO animal model with an easily controllable valve. Data showed that the serum creatinine transiently went up and down in the early days after obstruction and remained steady down in the following day, because contralateral kidney might compensate. The renal pelvis volume increased gradually after obstruction, while the renal cortical thickness decreased. Significant difference between the sham control group and PUUO group was found on the 10th day after obstruction. The pathological examination also demonstrated that the pathophysiologic morphology of obstruction had been achieved, while the sham operation did not harm the animal kidney. This result indicates the reliability and success of our model. After the relief of the obstruction, ultrasonographic and pathological examination demonstrated that the morphological and pathological changes would be partially reversed.

Several experimental models have been designed to create partial ureteral obstruction. Some of these were partial ligation of the ureter with sutures, which might lead to ureteral necrosis.^6^ Ulm and Miller firstly reported their models by embedding the ureter into the psoas muscle. However, those methods could not design the obstruction degree measurably and reversibly, and a second aggressive operation was needed to relieve the obstruction. Cheng and Chevalier used different tubes on the surrounding of the ureter to make PUUO models.^4^^,^^5^ Similar to Shokeir’s work, Algood made an insertion of artificial calculi via a vesicostomy or an ureterostomy.^8^^,^^9^^,^^10^ Beta irradiation on the ureter and cellophane bands has also been used to for the PUUO model. This ureteral obstruction might be irreversible because of the inflammation and adhesion. Ryan and Fitzpatrick introduced a new variable experimental PUUO model by inserting a stent into the midureter, in which the degree of obstruction was controlled by stents’ diameters.^11^ None of the previous procedures could simultaneously provide variable and measurable obstruction and easy obstructive relief at the same time for PUUO model.

Our procedure uses F6 ureteral catheters and tee joint valve. This procedure was proved to induce and relieve hydronephrosis as judged by morphological and pathological changes of the kidney. Different obstructive degrees can be simulated accurately and standardized by controlling the tee joint valve. Without a second operation, the obstruction is relieved through the tee joint valve. Moreover tee joint value allows easy collection of urine samples for studying the effect of electrolytes in urine in diseases and recovery phases. This experiment is relatively less traumatic, hence well-tolerated by animals, allowing for higher survival rates. Thus, our procedure could greatly reduce experimental variations and offer homogenous systemic conditions. This reversible PUUO model will be a useful tool for basic and clinical research in the etiology, development, prophylaxis, and treatment of obstructive nephropathy.

### Ethical approval

All applicable international, national, and/or institutional guidelines for the care and use of animals were followed. All procedures performed in studies involving animals were carried out in strict accordance with the recommendations in the Guide for the REGULATIONS FOR THE ADMINISTRATION OF AFFAIRS CONCERNING EXPERIMENTAL ANIMALS (Approved by the State Council on October 31, 1988 and promulgated by Decree No. 2 of the State Science and Technology Commission on November 14, 1988) and the Measures For The Administration Of Experimental Animals In Jiangsu (Serial number: 45) Province Decree of Jiangsu Provincial Government. The protocol was approved by the Committee on the Ethics of Animal Experiments of the Drum tower hospital affiliated to Medical School of Nanjing University. All surgeries were performed under ketamine intramuscular anesthesia, and all efforts were made to minimize animal suffering.
